# Hong Kong Urological Association–Hong Kong Society of Uro-Oncology 2025 consensus on the management of locally advanced or metastatic renal cell carcinoma

**DOI:** 10.3389/fonc.2026.1799885

**Published:** 2026-04-23

**Authors:** Darren M. C. Poon, Philip W. K. Kwong, Samson Y. S. Chan, Brian S. H. Ho, Martin H. C. Lam, K. S. Law, Angus K. C. Leung, Clarence L. H. Leung, David K. W. Leung, W. K. Ma, Barry B. W. Wo, Kenneth C. W. Wong, Philip Y. Wu, Peter K. F. Chiu

**Affiliations:** 1 Department of Clinical Oncology, State Key Laboratory of Translational Oncology, Sir YK Pao Centre for Cancer, Hong Kong Cancer Institute, The Chinese University of Hong Kong, Hong Kong, Hong Kong SAR, China; 2 Comprehensive Oncology Centre, Hong Kong Sanatorium and Hospital, Hong Kong, Hong Kong SAR, China; 3 Hong Kong Integrated Oncology Centre, Hong Kong, Hong Kong SAR, China; 4 S.H. Ho Urology Centre, Department of Surgery, The Chinese University of Hong Kong, Hong Kong, Hong Kong SAR, China; 5 Minimal Access Urology, Hong Kong, Hong Kong SAR, China; 6 Department of Clinical Oncology, Princess Margaret Hospital, Hong Kong, Hong Kong SAR, China; 7 AMO Oncology Centre, Hong Kong, Hong Kong SAR, China; 8 eFLO Urology, Hong Kong, Hong Kong SAR, China; 9 Hong Kong Urology Clinic, Hong Kong, Hong Kong SAR, China; 10 Department of Clinical Oncology, Tuen Mun Hospital, Hong Kong, Hong Kong SAR, China; 11 Department of Clinical Oncology, The Chinese University of Hong Kong, Hong Kong, Hong Kong SAR, China; 12 Department of Clinical Oncology, Pamela Youde Nethersole Eastern Hospital, Hong Kong, Hong Kong SAR, China

**Keywords:** adjuvant pembrolizumab, cytoreductive nephrectomy, metastasectomy, metastasis-directed therapy, stereotactic body radiation therapy, tyrosine kinase inhibitor

## Abstract

**Background:**

Continuous efforts have been made to optimise treatment approaches for renal cell carcinoma (RCC), including locally advanced (la)RCC and metastatic (m)RCC. To update their joint consensus statements on the management of la/mRCC, the Hong Kong Urological Association and the Hong Kong Society of Uro-Oncology convened a series of Delphi meetings.

**Methods:**

A panel of six urologists and eight clinical oncologists was formed, addressing five areas: (i) surgical and (neo)adjuvant treatment in laRCC; (ii) first-line management of clear cell mRCC; (iii) second- and later-line treatment of clear cell mRCC; (iv) management of mRCCs with sarcomatoid features or non-clear cell histology; and (v) management of oligometastatic RCC. Consensus statements were drafted based on an extensive literature review and panel discussions. A consensus statement was established only if ≥ 80% of the panellists selected ‘accept completely’ or ‘accept with some reservation’ from a five-point Likert scale through anonymous voting.

**Results:**

A total of 42 consensus statements were accepted. Compared with the previous version, this set of consensus statements asserted the use of adjuvant therapy for laRCC and the use of immunotherapy-based combination therapy across different types of mRCC; multiple new areas were also added, including active surveillance in mRCC patients with very favourable prognosis, a novel therapeutic agent for mRCC (belzutifan), and the definitions and management of oligometastatic/oligoprogressive RCC.

**Conclusion:**

While several research gaps remain to be addressed, this set of consensus statements reflects contemporary approaches for la/mRCC management, offering practical references for clinicians in Hong Kong and the Asia-Pacific region.

## 1 Introduction

Kidney cancer, predominantly renal cell carcinoma (RCC), is a prevalent malignancy worldwide (1). From 2000 to 2021, the global incidence of RCC increased by 0.15% on average annually, with an age-standardised incidence rate of 4.52 per 100, 000 population in 2021 (1). The largest increases in RCC incidence were observed in highly and moderately developed countries (1). In Hong Kong, the incidence of kidney and other urinary organ cancers, excluding the bladder, has been increasing over the past decade (2). In 2022, this cancer category ranked as the seventh most common among the male population of Hong Kong, with 562 new cases recorded and an incidence rate of 16.8 per 100, 000 males (2).

RCC prognosis is highly dependent on the disease stage at diagnosis (3). Approximately 80% of RCC cases are diagnosed at a localised stage, primarily treated with partial or radical nephrectomy, and > 90% of these patients are expected to survive up to 5 years (3). However, 20–40% of patients with localised RCC experience disease recurrence after surgery (4). Additionally, patients with locally advanced (la)RCC or metastatic (m)RCC have a worse prognosis, with 5-year cancer-specific survival rates ranging from 30–80% (3).

The aggressiveness of la/mRCC highlights an unmet medical need, requiring substantial efforts to optimise treatment strategies and improve patient outcomes. In 2021, the Hong Kong Urological Association (HKUA) and the Hong Kong Society of Uro-Oncology (HKSUO) jointly established a set of consensus statements and recommendations for the management of la/mRCC (5). Since then, the therapeutic paradigm for la/mRCC has rapidly evolved, with a growing body of evidence supporting multiple novel therapies, such as adjuvant immunotherapy (IO) for laRCC (6), a hypoxia-inducible factor 2 alpha inhibitor treatment (belzutifan) for clear cell mRCC (7), IO + tyrosine kinase inhibitor (TKI) combination therapy for non-clear cell mRCC (8, 9), and various local and systemic treatment modalities for oligometastatic RCC (10–13). Based on recent evidence, the two societies updated their joint consensus statements on the management of la/mRCC.

## 2 Methods

The HKUA and the HKSUO convened a consensus panel by inviting six member urologists and eight clinical oncologists based on their interest and experience in managing RCC patients in Hong Kong. The panel addressed la/mRCC management in five distinct parts: (i) surgical and (neo)adjuvant treatment in laRCC; (ii) first-line management of clear cell mRCC; (iii) second- and later-line treatment of clear cell mRCC; (iv) management of mRCC with sarcomatoid features or non-clear cell histology; and (v) management of oligometastatic RCC.

To lay the groundwork for panel discussions, a medical communications agency was commissioned to search for relevant literature on the PubMed database. The search terms included ‘locally advanced renal cell carcinoma’, ‘metastatic renal cell carcinoma’, ‘cytoreductive nephrectomy’, ‘neoadjuvant’, ‘adjuvant’, ‘monitoring’, ‘genomic’, ‘systemic therapy’, ‘sarcomatoid’, ‘non-clear cell’, ‘oligometastatic’, and ‘oligoprogressive’. Target article types included randomised controlled trials, observational studies, reviews, and clinical guidelines with a publication period between June 2021 and June 2025. The agency screened titles and abstracts for relevance, and the panel subsequently reviewed the full text of these papers.

Using the modified Delphi method (14), the panellists addressed each area of focus by presenting the sorted literature and other papers they deemed relevant, as well as sharing their clinical experiences in a series of meetings. Each topic was addressed by two to four panellists, followed by panel discussions. At the final meeting, the panel discussed, modified, and finalised draft statements derived from the meeting minutes. Each panellist voted on every statement via an online platform, assessing the practicability of the recommendation. A consensus statement was established only if ≥ 80% of the panellists selected ‘accept completely’ or ‘accept with some reservation’, from five possible responses, which also included ‘accept with major reservation’, ‘reject with reservation’, and ‘reject completely’. **Supplementary Appendix S1** shows the full voting records for each statement.

## 3 Results

Forty-two consensus statements were accepted (**Table 1**), with the rationale for each described below.

**Table 1:** All accepted consensus statements.

Part	Section/statement
1	Surgical and (neo)adjuvant treatment in locally advanced RCC
1.1	Role of CN
	1. Upfront CN can be considered in patients with: a. Advanced age (≥ 65 years); b. Severe kidney-related symptoms (e.g. severe haematuria); and/or c. Oligometastases or low metastatic burden.
	2. Deferred CN can be considered in selected patients who have received: a. IO-based combination therapy (IO+IO or IO+TKI); or b. TKI-based therapy.
1.2	Role of neoadjuvant therapy
	3. Routine use of neoadjuvant systemic therapy for locally advanced RCC is not recommended.
1.3	Role of adjuvant therapy
	4. Urologists should serve as a care coordinator and refer those with intermediate-to-high risk of recurrence and M1 NED to an oncologist to assess the need for adjuvant therapy.
	5. Adjuvant therapy should be initiated within 16 weeks after nephrectomy for eligible patients.
1.4	Monitoring of adjuvant therapy
	6. Before the start of adjuvant therapy, contrast-enhanced CT of the chest, abdomen, and pelvis is recommended.
	7. During adjuvant therapy, complete blood counts, liver function, and creatinine and thyroid-stimulating hormone levels should be checked regularly.
	8. In years 1 and 2 post adjuvant therapy, contrast-enhanced CT of the chest, abdomen, and pelvis every 3–6 months is recommended.
	9. In years 3–5 post adjuvant therapy, contrast-enhanced CT of the chest, abdomen, and pelvis every 6 months is recommended.
	10. After year 5 post adjuvant therapy, the frequency of imaging and duration of follow-up should be tailored to each patient’s risk of recurrence.
	11. PET-CT or MRI can be considered to assess cases with equivocal CT findings.
	12. MRI can be used if CT is contraindicated.
	13. Imaging of the bone or brain is recommended only when clinically indicated.
2	First-line management of metastatic clear cell RCC
2.1	Role of genomic testing
	1. Routine genomic testing to inform treatment selection for metastatic clear cell RCC is not recommended.
2.2	Management of patients with prior exposure to adjuvant pembrolizumab
	2. In patients who have metastatic recurrence during adjuvant pembrolizumab, TKI monotherapy is a treatment option.
	3. Rechallenging with IO+TKI may be considered in the metastatic setting in patients who have recurrence ≥ 12 months after the completion of adjuvant pembrolizumab.
2.3	Selection of first-line systemic therapy
	4. Active surveillance is a feasible management approach for selected patients (e.g. IMDC very-favourable risk, oligometastatic disease [refer to Part 5], low metastatic volume, or compliance with a follow-up schedule).
	5. In patients with IMDC very-favourable risk, TKI monotherapy is a treatment option.
	6. IO+TKI is the standard of care for IMDC favourable-risk patients.
	7. TKI monotherapy is an alternative regimen for selected patients with IMDC favourable risk (e.g. low metastatic burden and no symptoms).
	8. IMDC intermediate/poor-risk patients should be treated with IO+IO or IO+TKI.
	9. IO+TKI is the preferred regimen for IMDC intermediate/poor-risk patients with rapidly progressive or extensive disease.
	10. Treatment discontinuation can be considered in patients who have completed 2 years of IO+IO treatment.
2.4	Treatment monitoring
	11. To monitor treatment response, contrast-enhanced CT of the thorax, abdomen, and pelvis should be performed regularly (e.g. every 2–4 months).
	12. Imaging of regions outside the chest, abdomen, and pelvis should only be performed when clinically indicated.
3	Second- and later-line treatment of metastatic clear cell RCC
3.1	Second-line treatment
	1. IO rechallenge is not recommended in patients who have progressed on IO+IO or IO+TKI in the first-line metastatic setting.
	2. In patients who have progressed on IO+IO or IO+TKI in the first-line metastatic setting, subsequent treatment options include: a. Any TKI that has not previously been used; b. Lenvatinib + everolimus; and c. Lenvatinib + belzutifan.
	3. Belzutifan is a treatment option in patients who have been exposed to a TKI and an IO (in combination or sequentially).
	4. Patients who have progressed on first-line TKI monotherapy can be treated with: a. Cabozantinib; b. Nivolumab; c. Lenvatinib + everolimus; or d. IO+TKI.
3.2	Third-line treatment and beyond
	5. In patients exposed to an IO, a TKI, and belzutifan, lenvatinib + everolimus may be a treatment option.
4	Management of metastatic RCC with sarcomatoid features or non-clear cell histology
4.1	Management of patients with sarcomatoid features
	1. IO+IO or IO+TKI is recommended for IMDC intermediate/poor-risk patients with metastatic RCC and sarcomatoid dedifferentiation.
	2. IO+IO or IO+TKI is a treatment option in IMDC favourable-risk patients with metastatic RCC and sarcomatoid dedifferentiation.
4.2	Management of patients with non-clear cell RCC
	3. Preferred first-line treatment options for advanced non-clear cell RCC include: a. Pembrolizumab + lenvatinib; b. Nivolumab + cabozantinib; c. Nivolumab + ipilimumab; and d. Cabozantinib.
5	Management of oligometastatic RCC
5.1	Definitions of oligometastatic and oligoprogressive RCC
	1. Synchronous oligometastatic RCC should be defined as ≤ 3 metastatic lesions on contrast-enhanced CT at initial diagnosis.
	2. Metachronous oligometastatic RCC should be defined as ≤ 3 metastatic lesions on contrast-enhanced CT > 1 year after primary tumour diagnosis.
	3. Oligoprogressive RCC should be defined as progression involving ≤ 3 metastatic lesions, including new lesions and RECIST-defined enlargement of known lesions, on contrast-enhanced CT following systemic therapy.
5.2	Management of oligometastatic RCC
	4. Upfront systemic therapy, rather than MDT, is preferable in patients with oligometastatic RCC who have: a. IMDC poor risk; b. Brain metastases; c. Non-clear cell RCC; d. Sarcomatoid features; and/or e. Rapidly progressive disease within 6 months.
	5. In selected patients with oligometastatic RCC, viable treatment options include: a. Nephrectomy and complete MDT followed by active surveillance; b. Nephrectomy and complete MDT followed by adjuvant pembrolizumab; c. Nephrectomy and complete MDT followed by IO combinations; and d. IO-based combination therapy followed by consolidative nephrectomy and MDT in good responders.
	6. In oligometastatic RCC, metastasis-directed SBRT may be considered to delay both progression and the need for systemic therapy.
	7. In selected patients with oligometastatic RCC, the following treatment approaches can be considered: a. SBRT followed by active surveillance; b. SBRT followed by IO+TKI; and c. SBRT followed by IO+IO.
5.3	Management of oligorecurrent/oligoprogressive RCC
	8. In patients with oligorecurrent disease after nephrectomy: a. Systemic IO-based combination therapies should be the first-line treatment. b. MDT (e.g. SBRT) can be considered to delay progression.
	9. In patients with oligoprogressive metastases following nephrectomy and systemic IO-based therapy: a. SBRT to the new lesions followed by continuation of the current systemic therapy should be considered. b. MDT typically requires withholding IO/TKI therapy temporarily.

CN, cytoreductive nephrectomy; CT, computed tomography; IMDC, International Metastatic RCC Database Consortium; IO, immunotherapy; MDT, metastasis-directed therapy; MRI, magnetic resonance imaging; NED, no evidence of disease; PET, positron emission tomography; RCC, renal cell carcinoma; RECIST, Response Evaluation Criteria in Solid Tumours; SBRT, stereotactic body radiation therapy; TKI, tyrosine kinase inhibitor.

### 3.1 Surgical and (neo)adjuvant treatment in laRCC

#### 3.1.1 Role of cytoreductive nephrectomy (CN)

Statement 1. Upfront CN can be considered in patients with:

- Advanced age (≥ 65 years);
- Severe kidney-related symptoms (e.g. severe haematuria); and/or
- Oligometastases or low metastatic burden.

Statement 2. Deferred CN can be considered in selected patients who have received:

- IO-based combination therapy (IO+IO or IO+TKI); or
- TKI-based therapy.

In the TKI era, upfront CN was not considered to offer any additional survival benefits, based on two phase 3 randomised trials, CARMENA (15) and SURTIME (16). Notably, these studies mainly included patients with International mRCC Database Consortium (IMDC) poor risk and a high metastatic volume. Several panellists commented that IMDC poor-risk patients may be prone to distant failure; therefore, upfront CN with delayed systemic therapy may lead to inferior overall survival (OS).

The combined use of CN and IO has been investigated in retrospective studies only. A pooled analysis of patients with stage 4 mRCC at initial diagnosis (N = 981) from five phase 3 trials of IO+TKI (17) showed that upfront CN was associated with improved progression-free survival (PFS; median, 15 vs. 11 months; adjusted hazard ratio [HR], 0.71; 95% confidence interval [CI], 0.59–0.85), OS (median, 46 vs. 28 months; adjusted HR, 0.63; 95% CI, 0.51–0.77), and objective response rate (ORR, 60% vs. 46%). An analysis of the IMDC database included patients who received IO-based combination therapy with delayed CN (n = 24) or upfront CN (n = 182), or without CN (n = 179) (18). The receipt of CN (delayed or upfront) was an independent favourable prognostic factor for OS (HR, 0.45; 95% CI, 0.26–0.78; *P* = 0.005) (18). There were also multiple smaller studies demonstrating desirable oncological and safety outcomes of deferred CN following IO (19–21). However, the limitations of these retrospective studies included potential selection bias and immortal time bias. Despite the potential of CN for improving prognosis in the IO era, careful patient selection should be the prerequisite.

Based on the current evidence and panel discussions, upfront CN can be considered in patients with specific clinical features, such as severe kidney-related symptoms (e.g. severe haematuria, which may require hospitalisation and prevent delivery of systemic therapy) or oligometastases/low metastatic burden, with advanced age (≥ 65 years) not being a restriction. In contrast, the panel did not agree to use upfront CN in patients with the following characteristics: Eastern Cooperative Oncology Group performance status ≥ 2, IMDC intermediate/poor-risk disease, a small primary tumour (≤ 4 cm), sarcomatoid dedifferentiation, non-clear cell histology, or rapidly progressive disease. These factors were considered to possibly represent unfavourable resectability or prognosis (22), justifying the upfront use of systemic therapy.

Additionally, the panel agreed that deferred CN can be considered in selected patients who have received upfront systemic therapy with IO-based combinations or TKI alone. A good response to systemic therapy (e.g. tumour shrinkage) is a favourable factor for deferred CN (22).

#### 3.1.2 Role of neoadjuvant therapy

Statement 3. Routine use of neoadjuvant systemic therapy for laRCC is not recommended.

laRCC features a primary tumour with venous tumour thrombus (involving the renal vein and/or the inferior vena cava; levels I–IV), invasion beyond Gerota’s fascia (T4 disease), and/or bulky regional lymphadenopathy (N1/2 disease), which is marginally resectable due to its proximity to critical structures (23). Neoadjuvant therapy for laRCC is aimed at downstaging the tumour/thrombus, facilitating complete resection, and treating micrometastases early, with the potential for informing the choice of subsequent systemic therapy (24). Multiple TKIs and IOs, alone or in combination, as neoadjuvant therapy for laRCC have demonstrated tumour downsizing and partial responses; however, the major caveats of related studies include a single-arm design and small sample sizes (24, 25).

The phase 3 randomised PROSPER ECOG-ACRIN EA8143 trial investigated perioperative nivolumab therapy for laRCC (26). The participants were randomised to receive surgery alone or surgery plus nivolumab (one dose preoperatively and nine doses postoperatively). The study was discontinued at a planned interim analysis because of futility; the primary endpoint of improvement in recurrence-free survival (RFS) was not met (HR, 0.95; 95% CI, 0.74–1.22; one-sided *P* = 0.34). A similar result was observed in the prespecified sensitivity analysis in which patients who did not receive surgery or were not disease-free after surgery were censored at Day 1 instead of being counted as events at Day 1.

Multiple studies are investigating IO+TKI combinations as neoadjuvant therapy for laRCC, such as pembrolizumab + lenvatinib (NCT05319015, NCT05733715, and NCT04393350), pembrolizumab + axitinib (NCT05969496), and avelumab + axitinib (NCT03341845).

Currently, there is no conclusive evidence to support the benefit of neoadjuvant therapy for laRCC. Importantly, concerns about this approach include risks of treatment-related adverse events, disease progression due to surgery delay or preclusion, and surgical morbidities (24). Neoadjuvant therapy for laRCC is preferably limited to a clinical trial setting, in which the patient operability before and after systemic therapy should be assessed by a multidisciplinary team.

#### 3.1.3 Role of adjuvant therapy

Statement 4. Urologists should serve as a care coordinator and refer those with intermediate-to-high risk of recurrence and M1 with no evidence of disease (NED) to an oncologist to assess the need for adjuvant therapy.

Statement 5. Adjuvant therapy should be initiated within 16 weeks after nephrectomy for eligible patients.

The phase 3 randomised KEYNOTE-564 trial demonstrated that, at a median follow-up of ~57 months, adjuvant pembrolizumab therapy significantly improved disease-free survival (DFS; HR, 0.72; 95% CI, 0.59−0.87) and OS (HR, 0.62; 95% CI, 0.44−0.87; *P* = 0.005) compared with placebo in patients at intermediate-to-high risk for recurrence (**Table 2**) after surgical treatment for laRCC (6, 27). The DFS and OS benefits of adjuvant pembrolizumab were consistent across subgroups by metastatic staging, disease risk category, and sarcomatoid feature (6, 27).

**Table 2:** Key inclusion criteria of KEYNOTE-564 (27).

Characteristic category	Characteristics
Histology	Clear cell renal cell carcinoma
Surgical treatment	• Partial or radical nephrectomy; and • Synchronous or metachronous (within 1 year post nephrectomy) metastasectomy of any solid, isolated, soft-tissue, non-bone, non-brain metastatic lesions that could be resected completely with negative margins
Risk factors for disease recurrence	Intermediate to high risk • pT2 tumours with nuclear grade 4 or sarcomatoid features • pT3 tumours • N0M0
	High risk • pT4 tumours and N0M0 • pT_any_N1M0
	M1 no evidence of disease

Besides KEYNOTE-564, other phase 3 randomised trials on adjuvant IO for laRCC include PROSPER (perioperative nivolumab) (26), IMmotion010 (atezolizumab) (28), and CheckMate 914 (nivolumab + ipilimumab) (29); however, none of the regimens in these trials showed DFS benefits. There are several possible explanations for why KEYNOTE-564 was the only trial to yield a positive result (30). First, KEYNOTE-564 enrolled higher-risk patients (pT2 [high-grade], pT3/pT4, N+, or M1 NED) who likely benefited more from IO because of greater tumour antigen exposure and immune activation. Second, pembrolizumab, a programmed cell death 1 (PD-1) inhibitor, may offer stronger antitumour activity than atezolizumab, a programmed cell death ligand 1 (PD-L1) inhibitor, while having a more favourable tolerability profile than nivolumab + ipilimumab. Third, KEYNOTE-564 was well designed and adequately powered to detect a clinically meaningful treatment benefit. Fourth, RCCs are heterogeneous, and some may respond better to PD-1 inhibition.

Based on the results of KEYNOTE-564, international guidelines (31, 32) have recommended adjuvant pembrolizumab therapy as the standard of care for selected patients with laRCC post nephrectomy. Other regimens are being investigated in the adjuvant setting, including pembrolizumab + belzutifan in the phase 3 randomised LITESPARK-022 trial (33).

In Hong Kong, patients with laRCC are mainly treated by urologists and clinical oncologists, whose collaboration and referral mechanisms are important for optimising patient care. The panel agreed that urologists should serve as a care coordinator, referring potential patients to oncologists to assess the need for adjuvant pembrolizumab.

Additionally, based on the KEYNOTE-564 study design (27) and practical considerations, such as surgery recovery time and procedural applications for medical subsidies and pathology reports, the panel suggested that adjuvant pembrolizumab should be initiated within 16 weeks after nephrectomy for eligible patients.

#### 3.1.4 Monitoring of adjuvant therapy

Statement 6. Before the start of adjuvant therapy, contrast-enhanced computed tomography (CT) of the chest, abdomen, and pelvis is recommended.

Statement 7. During adjuvant therapy, complete blood counts, liver function, and creatinine and thyroid-stimulating hormone levels should be checked regularly.

Statement 8. In years 1 and 2 post adjuvant therapy, contrast-enhanced CT of the chest, abdomen, and pelvis every 3–6 months is recommended.

Statement 9. In years 3–5 post adjuvant therapy, contrast-enhanced CT of the chest, abdomen, and pelvis every 6 months is recommended.

Statement 10. After year 5 post adjuvant therapy, the frequency of imaging and duration of follow-up should be tailored to each patient’s risk of recurrence.

Statement 11. Positron emission tomography (PET)-CT or magnetic resonance imaging (MRI) can be considered to assess cases with equivocal CT findings.

Statement 12. MRI can be used if CT is contraindicated.

Statement 13. Imaging of the bone or brain is recommended only when clinically indicated.

The above statements on the post-adjuvant pembrolizumab follow-up schedule were adapted from KEYNOTE-564 (27) and recommendations from the U.S. National Comprehensive Cancer Network (32). It should be noted that 85% of recurrent RCC cases occur within 5 years after curative surgery (34). The most common recurrence sites include the lungs (50–60%) and lymph nodes (30–40%) (4, 34). Baseline and regular imaging of these sites is recommended, preferably using contrast-enhanced CT, or alternatively, PET-CT or MRI where applicable. Regular blood testing helps monitor for pembrolizumab-related toxicities.

In patients who are at low risk for recurrence and have remained recurrence-free for 2–3 years, de-intensifying the frequency of imaging may be considered (35). The duration of follow-up may also be individualised; in patients who are at high risk for recurrence, surveillance for > 5 years may be considered. Researchers have suggested that patients with high-risk histology be followed for 10 years (36).

### 3.2 First-line management of clear cell mRCC

#### 3.2.1 Role of genomic testing

Statement 1. Routine genomic testing to inform treatment selection for clear cell mRCC is not recommended.

Despite the frequent use of PD-L1 status as a stratification factor in randomised trials of IO-based combination therapies, its role in informing treatment selection is ambiguous. A meta-analysis revealed that high expression of PD-L1 was linked to better PFS outcomes with IO; however, no such association was observed regarding OS outcomes (37). In addition to PD-L1, a range of biomarkers, including angiogenesis, CD8^+^ T cells, myeloid inflammatory gene expression signatures, PD-1, T-cell immunoglobulin and mucin-domain containing-3, and lymphocyte activation protein-3, have shown correlation with PFS outcomes with IO. However, data on OS remain limited (38, 39). Tumour mutation and neoantigen burdens appear to have no impact on treatment responses (38). Studies are investigating the role of prevalent mutations in clear cell RCC (e.g. *VHL*) in IO response (40). Other emerging techniques to predict treatment responses in RCC, such as circulating tumour DNA analysis, single-cell RNA sequencing, and high-dimensional spatial analysis, warrant further verification (40).

#### 3.2.2 Management of patients with prior exposure to adjuvant pembrolizumab

Statement 2. In patients who have metastatic recurrence during adjuvant pembrolizumab, TKI monotherapy is a treatment option.

Statement 3. Rechallenging with IO+TKI may be considered in the metastatic setting in patients who have recurrence ≥ 12 months after the completion of adjuvant pembrolizumab.

In the adjuvant setting, pembrolizumab is the only IO to demonstrate a survival benefit supported by phase 3 randomised data. Increased use of this regimen is expected; therefore, management in the post-adjuvant pembrolizumab setting warrants discussion.

Data on IO rechallenge after adjuvant pembrolizumab remain limited. A retrospective study (41) showed that, in 94 patients with recurrent RCC following adjuvant IO (pembrolizumab, 39%; atezolizumab, 30%; and nivolumab + ipilimumab, 16%), first-line systemic therapy with TKI alone (39%), IO+TKI (28%), or IO+IO (13%) in the metastatic setting yielded similar PFS and OS rates, regardless of the prior adjuvant agent or disease-free interval (≤ 3 vs. > 3 months) following adjuvant therapy. Despite its small sample size, this study suggested that both TKI monotherapy and IO-based combination therapy may be considered to treat metastatic recurrence following adjuvant pembrolizumab. The panel agreed that TKI monotherapy is an option, but noted that IO+IO and IO+TKI should not be excluded in the post-adjuvant pembrolizumab setting. Additionally, the panel reached consensus that IO+TKI may be considered in patients with RFS ≥ 1 year after completing adjuvant pembrolizumab therapy. Consistently, the European Association of Urology recommended that IO rechallenge be considered in patients who experience recurrence ≥ 6 months post-adjuvant pembrolizumab (42).

Randomised controlled trials are evaluating regimens for IO-experienced patients, particularly belzutifan combined with agents such as pembrolizumab (43), cabozantinib (44), and lenvatinib (45). Results from these studies are anticipated to better inform treatment selection in patients who have received IO in the adjuvant or first-line metastatic setting.

#### 3.2.3 Selection of first-line systemic therapy

Statement 4. Active surveillance is a feasible management approach for selected patients (e.g. IMDC very-favourable risk, oligometastatic disease [refer to Part 3.5], low metastatic volume, or compliance with a follow-up schedule).

Statement 5. In patients with IMDC very-favourable risk, TKI monotherapy is a treatment option.

Statements 4–9 represent the panel’s proposed first-line systemic therapies for patients with clear cell mRCC across risk categories. **Table 3** summarises these suggestions.

**Table 3:** Proposed first-line systemic therapies for patients with clear cell metastatic renal cell carcinoma across different risk categories.

Risk category	IMDC very-favourable risk	IMDC favourable risk	IMDC intermediate/poor risk
Preferred regimens	Undetermined	Pembrolizumab + lenvatinib Pembrolizumab + axitinib Nivolumab + cabozantinib	Pembrolizumab + lenvatinib* Pembrolizumab + axitinib* Nivolumab + cabozantinib* Nivolumab + ipilimumab
Possible regimens	Active surveillance TKI monotherapy	TKI monotherapy (e.g. for patients with a low metastatic burden and no symptoms)	As above

*Immunotherapy plus tyrosine kinase inhibitor (TKI) is the preferred regimen for International Metastatic Renal Cell Carcinoma Database Consortium (IMDC) intermediate/poor-risk patients with rapidly progressive or extensive disease.

To further investigate the role of IO in IMDC favourable-risk patients, researchers have recently identified a subgroup of “very favourable-risk” patients (46, 47), highlighting the potential of more personalised treatment approaches. This subgroup is defined by a primary diagnosis to systemic therapy timeline ≥ 3 years, no brain, liver or bone metastases, and a 90% or 100% Karnofsky performance status (46, 47). Preliminary retrospective data showed that IO+IO was associated with a significant reduction in 2-year OS (HR, 3.19) compared with TKI alone in very favourable-risk patients (47), suggesting that less intensive or more tolerable therapy may be considered in this patient subgroup. The panel agreed that TKI monotherapy can be considered in very favourable-risk patients, while active surveillance can also be considered in those with an even lower risk (e.g. only oligometastatic disease and a low metastatic volume) (48). Notably, the very favourable-risk category is an emerging notion derived from retrospective data; further prospective studies are warranted to validate the clinical significance of this novel risk stratification.

Statement 6. IO+TKI is the standard of care for IMDC favourable-risk patients.

Statement 7. TKI monotherapy is an alternative regimen for selected patients with IMDC favourable risk (e.g. low metastatic burden and no symptoms).

Statement 8. IMDC intermediate/poor-risk patients should be treated with IO+IO or IO+TKI.

Statement 9. IO+TKI is the preferred regimen for IMDC intermediate/poor-risk patients with rapidly progressive or extensive disease.

Statement 10. Treatment discontinuation can be considered in patients who have completed 2 years of IO+IO treatment.

Compared with TKI alone, IO+TKI consistently demonstrated significant PFS (HRs, 0.47–0.69) and OS (HRs, ~0.70) benefits among intention-to-treat populations in pivotal phase 3 trials during extended follow-up (49–51). Notably, the relative OS benefits (a secondary endpoint) of IO+TKI appeared to diminish gradually in the long term (≥ 60 months post-treatment). By indirect comparison of IO+TKI regimens among patients with IMDC favourable-risk disease, pembrolizumab + lenvatinib appeared to offer the best PFS benefit (HR, 0.50; 95% CI, 0.35–0.71 vs. HR, 0.76; 95% CI, 0.57–1.02 for pembrolizumab + axitinib and HR, 0.69; 95% CI, 0.48–1.00 for nivolumab + cabozantinib).

IO+IO (nivolumab + ipilimumab) consistently showed significant PFS and OS benefits in patients with IMDC intermediate/poor-risk disease compared with TKI monotherapy during long-term follow-up of the phase 3 CheckMate 214 study (52). Among study populations with IMDC intermediate/poor-risk disease, IO+TKI appeared to offer higher ORRs (55–69%) and lower rates of progressive disease (6–11%) by indirect comparison with IO+IO, but IO+IO still yielded a long median OS and durable responses.

Recently, the multinational retrospective ARON-1 study analysed the outcomes of first-line IO+IO (n = 313) and IO+TKI (n = 416) in real-world patients with mRCC (86% clear cell) (53). In the subgroup of 616 patients with IMDC intermediate/poor-risk disease, IO+TKI was associated with a significantly longer median OS compared with IO+IO (55.7 vs. 29.7 months; *P* = 0.045) (53). In the overall population, IO+TKI was associated with a significantly higher rate of overall clinical benefit (complete response + partial response + stable disease) compared with IO+IO (84% vs. 72%; *P* < 0.001) (53). A separate analysis of the favourable-risk subgroup (N = 524) of the original study showed that, compared with sunitinib monotherapy, IO+TKI was associated with a significantly longer median PFS (30.7 vs. 22.9 months; *P* = 0.007) but a similar median OS (not reached for either group; *P* = 0.761) after a median follow-up of 37 months (54).

Multiple international guidelines (32, 42, 55, 56) have recommended IO-based combination therapies for patients with clear cell mRCC across all IMDC risk categories, while TKI monotherapy remains a treatment option in IMDC favourable-risk patients.

The panel agreed that IMDC favourable-risk patients should preferably be treated with IO+TKI [instead of TKI monotherapy as per the previous consensus (5)] or alternatively with TKI monotherapy (e.g. in asymptomatic patients with a low metastatic burden). The panel noted that long-term OS benefits of IO+TKI in favourable-risk patients appeared to be dubious in subgroup analyses of the pivotal trials, whereas IO+IO did not improve PFS or OS in this subgroup. The current consensus recommends all IO-based regimens for IMDC intermediate/poor-risk patients, in contrast to the previous guideline (5), which only endorsed IO+IO and pembrolizumab + axitinib. Additionally, the panel preferred IO+TKI over IO+IO in intermediate/poor-risk patients with rapidly progressive or extensive disease.

Regarding treatment discontinuation, the panel suggested a treatment course of nivolumab + ipilimumab of up to 2 years, particularly in patients with a complete response, which aligns with the CheckMate 214 study protocol (52). However, the panel did not reach consensus on when to stop IO+TKI treatment. In the pivotal trials on IO+TKI (49–51), IO discontinuation was planned at 2 years, whereas TKI was expected to continue until progression or unacceptable toxicity. Whether TKI can be discontinued in the IO+TKI setting remains to be determined.

#### 3.2.4 Treatment monitoring

Statement 11. To monitor treatment response, contrast-enhanced CT of the thorax, abdomen, and pelvis should be performed regularly (e.g. every 2–4 months).

Statement 12. Imaging of regions outside the chest, abdomen, and pelvis should only be performed when clinically indicated.

In line with the European Society for Medical Oncology guidelines (55), the panel agreed that contrast-enhanced CT of the thorax, abdomen, and pelvis should be performed regularly (e.g. every 2–4 months) to monitor response to systemic therapy. Additionally, the panel considered that imaging of other regions (using contrast-enhanced CT, PET-CT, or MRI where applicable) should only be performed when clinically indicated (e.g. presence of symptoms). Separately, the panel discussed a range of care approaches for common side effects related to IO and TKI (**Supplementary Appendix S2**) (57, 58). They noted that regular blood testing and physical examinations are required for toxicity monitoring in patients on systemic therapy.

The panel discussed whether continuous monitoring is necessary in patients who have stopped treatment, including those who have achieved a complete response. Several panellists noted that current guidelines and protocols do not address such requirements. They also shared that, in clinical experience, the number of such patients was limited, with most becoming lost to follow-up. The panel did not achieve consensus on monitoring these patients.

### 3.3 Second- and later-line treatment of clear cell mRCC

#### 3.3.1 Second-line treatment

Statement 1. IO rechallenge is not recommended in patients who have progressed on IO+IO or IO+TKI in the first-line metastatic setting.

The choice of second- and later-line treatment mainly depends on the systemic therapy previously used in the metastatic setting (**Table 4**). Two phase 3 randomised trials, CONTACT-03 [cabozantinib ± atezolizumab (59)] and TiNivo-2 [tivozanib ± nivolumab (60)], showed that second-line TKI alone and IO+TKI did not significantly differ in survival outcomes among patients who had progressed on first-line IO-based therapies for mRCC. Therefore, the panel agreed that IO rechallenge is not recommended for such patients.

**Table 4:** Proposed second- and later-line systemic therapies for clear cell metastatic renal cell carcinoma.

Previously used agents	Subsequent treatment options
IO+IO or IO+TKI	Any TKI that has not previously been used Lenvatinib + everolimus Lenvatinib + belzutifan
TKI alone	Cabozantinib Nivolumab Lenvatinib + everolimus IO+TKI
IO and TKI (in combination or sequentially)	Belzutifan
IO, TKI, and belzutifan	Lenvatinib + everolimus

IO, immunotherapy; TKI, tyrosine kinase inhibitor.

Statement 2. In patients who have progressed on IO+IO or IO+TKI in the first-line metastatic setting, subsequent treatment options include:

- Any TKI that has not previously been used;
- Lenvatinib + everolimus; and
- Lenvatinib + belzutifan.

Statement 3. Belzutifan is a treatment option in patients who have been exposed to a TKI and an IO (in combination or sequentially).

Several prospective studies provide insights into treatment strategies for patients who have progressed on IO-based therapy in the metastatic setting. In the phase 2 randomised LenCabo trial (61), lenvatinib + everolimus (n = 40) significantly improved the median PFS (15.7 vs. 10.2 months; HR, 0.51; 95% CI, 0.29–0.89; *P* = 0.02) compared with cabozantinib (n = 46) in participants, of whom 70% received IO+IO in the first-line setting. The phase 3 randomised LITESPARK-011 trial is investigating lenvatinib + belzutifan versus cabozantinib in patients who have received IO-based therapy in the first/second-line metastatic setting or as adjuvant therapy, with PFS and OS as dual primary endpoints (62). A press release (45) reported that, at a prespecified interim analysis, lenvatinib + belzutifan significantly improved PFS, but not OS, compared with cabozantinib; however, these preliminary findings should be interpreted cautiously. In the phase 3 randomised LITESPARK-005 trial of patients who had received prior IO and TKI (50% exposed to only one TKI) as monotherapy or combination therapy in any line of treatment, belzutifan (n = 374) significantly improved the rate of PFS at 18 months (24.0% vs. 8.3%; *P* = 0.002) compared with everolimus (n = 372), whereas 18-month OS rates in both groups (55.2% vs. 50.6%; HR, 0.88; 95% CI, 0.73–1.07; *P* = 0.20) did not significantly differ (7).

Based on the above evidence, the panel recommended that lenvatinib + everolimus, lenvatinib + belzutifan, and belzutifan monotherapy be considered as second-line treatment regimens in patients who have progressed on first-line IO+IO or IO+TKI, with some panellists noting that the use of lenvatinib + everolimus may be limited by its toxicity. Additionally, they agreed that any TKI that has not previously been used can be considered in patients exposed to prior IO+TKI, consistent with multiple international guidelines (32, 42, 55, 56).

Statement 4. Patients who have progressed on first-line TKI monotherapy can be treated with:

- Cabozantinib;
- Nivolumab;
- Lenvatinib + everolimus; or
- IO+TKI.

For patients who progress on first-line TKI monotherapy, the previous consensus recommended nivolumab or cabozantinib in the second-line setting (5). In this update, the panel now adds lenvatinib + everolimus and IO+TKI as additional considerations in these patients. The lenvatinib + everolimus regimen was supported by a phase 2 randomised trial that showed its PFS benefit relative to everolimus alone (median, 14.6 vs. 5.5 months; HR, 0.40; 95% CI, 0.24–0.68; *P* = 0.0005) in patients who had progressed on TKI monotherapy (63). Based on preliminary data from a real-world retrospective study (64), second-line IO+TKI in the post-TKI setting may offer a similar OS to first-line IO+TKI in IMDC intermediate/poor-risk patients with clear cell mRCC.

#### 3.3.2 Third-line treatment and beyond

Statement 5. In patients exposed to an IO, a TKI, and belzutifan, lenvatinib + everolimus may be a treatment option.

In a later-line setting, the panel agreed upon the use of belzutifan or lenvatinib + everolimus. The LITESPARK-005 trial (7) suggested that belzutifan can be used in patients who have received TKI and IO consecutively or in combination (Refer to Statement 3). Patients exposed to TKI, IO, and belzutifan are not well studied and have limited treatment options in clinical practice. Based on their expert insights, the panel suggested that lenvatinib + everolimus may be considered in these patients.

### 3.4 Management of mRCCs with sarcomatoid features or non-clear cell histology

#### 3.4.1 Management of patients with sarcomatoid features

Statement 1. IO+IO or IO+TKI is recommended for IMDC intermediate/poor-risk patients with mRCC and sarcomatoid dedifferentiation.

Statement 2. In IMDC favourable-risk patients with mRCC and sarcomatoid dedifferentiation, IO+IO or IO+TKI is a treatment option.

**Table 5** summarises Statements 1–3 of Part 3.4, representing the panel’s proposed treatment approaches for clear cell mRCC with sarcomatoid features and non-clear cell mRCC.

**Table 5:** Proposed systemic therapies for clear cell metastatic renal cell carcinoma (mRCC) with sarcomatoid features and non-clear cell mRCC.

Type of mRCC	Clear cell mRCC with sarcomatoid features		Non-clear cell mRCC
	IMDC favourable risk	IMDC intermediate/poor risk	
Preferred regimens	Undetermined	Pembrolizumab + lenvatinib Pembrolizumab + axitinib Nivolumab + cabozantinib Nivolumab + ipilimumab	Pembrolizumab + lenvatinib Nivolumab + cabozantinib Nivolumab + ipilimumab Cabozantinib
Possible regimens	Pembrolizumab + lenvatinib Pembrolizumab + axitinib Nivolumab + cabozantinib Nivolumab + ipilimumab	As above	As above

IMDC, International Metastatic RCC Database Consortium.

Sarcomatoid dedifferentiation may be associated with epithelial–mesenchymal transition, which can lead to carcinogenesis and may exacerbate tumour aggressiveness (65). Instead of a distinct RCC subtype, sarcomatoid RCC refers to RCC of any histology with the development of sarcomatoid features due to dedifferentiation (66). Up to 5% of RCCs of any histology have sarcomatoid features, while > 80% of sarcomatoid RCC cases have clear cell histology (67). Sarcomatoid RCC is frequently associated with mutations in the *TP53, BAP1, CDKN2A, MYC, PD-1*, and *PD-L1* genes (66).

Despite the lack of studies dedicated to patients with sarcomatoid RCC, subgroup analyses of multiple phase 3 randomised trials with long-term follow-up consistently demonstrated that first-line IO-based combination therapies (nivolumab + ipilimumab, pembrolizumab + lenvatinib, pembrolizumab + axitinib, and nivolumab + cabozantinib) significantly improved OS, PFS, and ORR compared with TKI monotherapy in patients with clear cell mRCC and sarcomatoid features (68–72). The multicentre retrospective ARON-1 study, which collected global experiences using IO-based combination therapies in patients with mRCC, showed that those with sarcomatoid features (n = 226) who received first-line IO+IO (55%) or IO+TKI (45%) had similar OS (median, 26.4 vs. 34.4 months; *P* = 0.729), PFS (median, 12.3 vs. 12.4 months; *P* = 0.606), and ORR (both 48%) (73).

The panel noted that, as more robust evidence emerges, the recommendation level for first-line IO-based combination therapy in clear cell mRCC with sarcomatoid features should be raised compared with the previous consensus (5), in line with recent international guidelines (42, 74).

Notably, most study patients with sarcomatoid mRCC had clear cell histology and IMDC intermediate or poor risk, which is consistent with routine clinical practice. There is strong evidence to support the use of IO-based combination therapies in these patients. Extrapolated from current evidence, the panel recommended that IO-based combination therapies be considered in patients with IMDC favourable-risk disease and sarcomatoid features, although this patient subgroup is not well studied.

#### 3.4.2 Management of patients with non-clear cell mRCC

Statement 3. Preferred first-line treatment options for advanced non-clear cell RCC include:

- Pembrolizumab + lenvatinib;
- Pembrolizumab + axitinib;
- Nivolumab + cabozantinib;
- Nivolumab + ipilimumab; and
- Cabozantinib.

Approximately 25% of RCCs are classified as non-clear cell RCC, with papillary RCC being the most common subtype, followed by chromophobe RCC, collecting duct RCC, renal medullary carcinoma, translocation RCC, and others (75). Several international guidelines (32, 55) make dedicated treatment recommendations for individual non-clear cell RCC subtypes. To streamline treatment decision-making, the panel formulated a general suggestion for patients with non-clear cell mRCC, irrespective of subtype. This approach aligns with the available evidence, which is mostly derived from studies of pooled patients with different non-clear cell RCC subtypes. In contrast to the previous recommendation of TKI monotherapy (cabozantinib or sunitinib) for non-clear cell mRCC (5), this update recommends first-line IO+TKI or cabozantinib, based on recent evidence.

Based on indirect comparison of updated results from larger-scale phase 2 trials (**Table 6**), including SWOG 1500 (76), KEYNOTE-B61 (77), NCT03635892 (78), and SUNNIFORECAST (79), first-line IO+TKI (pembrolizumab + lenvatinib or nivolumab + cabozantinib) appears to offer better OS, PFS, and ORR outcomes compared with TKI (cabozantinib) monotherapy and IO+IO (nivolumab + ipilimumab) in patients with non-clear cell mRCC. Additionally, assessment of pembrolizumab + lenvatinib in a larger sample (77) appeared to show longer median OS and PFS compared with nivolumab + cabozantinib (78). Notably, no head-to-head trials have been conducted to compare these regimens.

**Table 6:** Efficacy outcomes across systemic therapies in patients with non-clear cell metastatic renal cell carcinoma in the ARON-1 study (9).

Regimen	Pembrolizumab + lenvatinib	Pembrolizumab + axitinib	Nivolumab + cabozantinib	Nivolumab + ipilimumab	*P* value
Median OS (months)	31.9	31.2	27.8	24.6	0.651
2-yr OS rate (%)	69	61	54	51	0.047
Median PFS (months)	24.5	13.4	18.4	6.8	0.038
1-yr PFS rate (%)	66	57	63	35	< 0.001
ORR (%)	59	35	40	32	0.005

ORR, objective response rate; OS, overall survival; PFS, progression-free survival.

A systematic review and meta-analysis of 23 studies that investigated first-line treatments across various subtypes of non-clear cell mRCC showed that IO+TKI appeared to improve OS, PFS, and ORR outcomes compared with IO or TKI monotherapy (8). Real-world evidence from the global retrospective ARON-1 study (9) showed that first-line IO+TKI, particularly pembrolizumab + lenvatinib, was associated with superior clinical outcomes compared with IO+IO (**Table 7**) in patients with non-clear cell mRCC.

**Table 7:** Study features and efficacy outcomes from selected phase 2 trials on the treatment of metastatic non-clear cell renal cell carcinoma (nccRCC).

Study	SWOG 1500 (76)	KEYNOTE-B61 (77)	NCT03635892 (78)	SUNNIFORECAST (79)
Regimen	Cabozantinib	Pembrolizumab + lenvatinib	Nivolumab + cabozantinib	Nivolumab + ipilimumab
Study design	Phase 2 RCT*	Phase 2 single arm	Phase 2 single arm	Phase 2 RCT^†^
Line of treatment	1L or 2L	1L	1L or 2L	1L
Sample size	44	158	40	157
nccRCC histology (%)				
Papillary	100	59	80	56
Chromophobe	0	18	0	17
Collecting duct	0	0	0	4
Medullary	0	0	0	1
Others	0	10	5	22
Unclassified	0	13	15	0
Median follow-up (months)	17.5	22.8	34	21.5
Median OS (months)	21.5	NR	28 (1L + 2L)	33.2
18-mo OS rate (%)	~60	~70	70 (1L + 2L)	67
Median PFS (months)	7	17.9	11 (1L); 13 (2L)	5.4
ORR (%)	23	51	54 (1L); 36 (2L)	33

1L, first line; 2L, second line; ORR, objective response rate; OS, overall survival; PFS, progression-free survival; RCT, randomised controlled trial.

*Treatment arms included sunitinib, cabozantinib, crizotinib, and savolitinib; only data on cabozantinib, the most favourable therapy in the study, are shown.

^†^ The control arm was standard of care (sunitinib, 78%; cabozantinib, 7%; others, 15%); only data on nivolumab + ipilimumab, the superior therapy in the study, are shown.

Smaller-scale studies of systemic therapies for specific non-clear cell RCC subtypes include durvalumab + savolitinib for papillary RCC (28), TKI+TKI for chromophobe RCC (80), cabozantinib monotherapy for collecting duct RCC (81), gemcitabine + doxorubicin or bevacizumab + erlotinib for renal medullary carcinoma (82, 83), and axitinib + nivolumab for translocation RCC (84). Research to inform the optimal therapy for non-clear cell RCC based on varied biomarkers is ongoing (85).

Based on current evidence, the panel recommended pembrolizumab + lenvatinib, nivolumab + cabozantinib, nivolumab + ipilimumab, and cabozantinib monotherapy for patients with advanced non-clear cell RCC. Pembrolizumab + axitinib was not endorsed because of a lack of prospective data.

### 3.5 Management of oligometastatic RCC

#### 3.5.1 Definitions of oligometastatic and oligoprogressive RCC

Statement 1. Synchronous oligometastatic RCC should be defined as ≤ 3 metastatic lesions on contrast-enhanced CT at initial diagnosis.

Statement 2. Metachronous oligometastatic RCC should be defined as ≤ 3 metastatic lesions on contrast-enhanced CT > 1 year after primary tumour diagnosis.

Statement 3. Oligoprogressive RCC should be defined as progression involving ≤ 3 metastatic lesions, including new lesions and the Response Evaluation Criteria in Solid Tumours-defined enlargement of known lesions, on contrast-enhanced CT following systemic therapy.

Defining oligometastatic disease is important to identify a subgroup of patients with potentially better prognosis due to a lower number of metastatic lesions and thus requiring more personalised treatment, often through a multidisciplinary, multimodality approach (30, 86). In studies on metastasis-directed therapy (MDT; metastasectomy or stereotactic body radiotherapy [SBRT]) for oligometastatic disease, inclusion criteria typically allowed up to three to five lesions (30, 86). However, most study participants who derived from survival or local control benefits had ≤ 3 metastases treated with MDT. Accordingly, the panellists recommended defining oligometastatic and oligoprogressive RCC as ≤ 3 metastatic lesions, rather than ≤ 5, to better reflect the clinical applicability of MDT and to prompt timely systemic therapy for patients with more extensive disease.

In addition to the number of lesions, the timing of metastasis development is an important factor in treatment decision-making for patients with oligometastatic disease (30). Overall, 25–30% of patients with RCC present with metastases at diagnosis (synchronous mRCC), while another 25–30% of patients develop metastases after surgery (e.g. > 1 year after nephrectomy) for localised disease (metachronous mRCC) (87). Patients may also develop new lesions or experience progression or enlargement of previous lesions following systemic treatment (86). With advances in systemic therapies improving initial disease control and prolonging treatment-free intervals, cases of oligoprogression have become increasingly common in recent years. Management of oligometastatic or oligoprogressive RCC should also consider the location of metastasis, IMDC risk stratification, and response to prior therapy (e.g. adjuvant therapy) (30).

#### 3.5.2 Management of oligometastatic RCC

Statement 4. Upfront systemic therapy, rather than MDT, is preferable in patients with oligometastatic RCC who have:

- IMDC poor risk;
- Brain metastases;
- Non-clear cell RCC;
- Sarcomatoid features; and/or
- Rapidly progressive disease within 6 months.

There is a lack of evidence to support the benefits of local therapy in patients with oligometastatic RCC who have the above risk factors for tumour aggressiveness and disease progression. The panel recommended that upfront systemic therapy be prioritised in these patients, in line with treatment approaches for patients with poly-metastatic RCC (Refer to 3.2.3, 3.4.1, and 3.4.2).

Statement 5. In selected patients with oligometastatic RCC, viable treatment options include:

- Nephrectomy and complete MDT followed by active surveillance;
- Nephrectomy and complete MDT followed by adjuvant pembrolizumab;
- Nephrectomy and complete MDT followed by IO+IO or IO+TKI; and
- IO+IO or IO+TKI followed by consolidative nephrectomy and MDT in good responders.

A systematic review (88) found that complete metastasectomy was associated with improved OS or cancer-specific survival (median, 40.8 vs. 14.8 months) and symptom relief (e.g. pain management in bone metastases) compared with incomplete or no metastasectomy in patients with mRCC. The most studied surgical sites were the lungs, bone, and liver (88). Factors associated with favourable outcomes following metastasectomy included a good performance status, IMDC favourable and intermediate risk, solitary or oligometastatic lesions, metachronous metastasis, the absence of sarcomatoid features, clear cell histology, and complete resection (88). Another systematic review (89) further demonstrated that metastasectomy for lung lesions may be associated with better survival outcomes compared with metastasectomy for lesions at other sites. Notably, all studies on metastasectomy in mRCC were retrospective.

As discussed in Statement 4 of Part 3.2.3, active surveillance can be considered in selected patients with mRCC and a very favourable prognosis to delay the use of systemic therapy and corresponding morbidities (48). In line with this, the panellists agreed that active surveillance can be considered in selected patients with oligometastatic RCC who have undergone nephrectomy and complete MDT.

Complete metastasectomy can achieve M1 NED (90), creating an opportunity for selected patients to receive adjuvant pembrolizumab and potentially prolong DFS, as supported by KEYNOTE-564 (although only 5.8% of participants had M1 NED, all underwent metastasectomy within 1 year after nephrectomy) (6, 27). Randomised placebo-controlled trials showed that adjuvant therapy with pazopanib (91), atezolizumab (28), or sorafenib (92) did not improve DFS or RFS in patients with M1 NED after metastasectomy.

CN may help reduce tumours with resistant clones, halt metastatic seeding, and modulate immune systems (93). As noted in Part 3.1.1, upfront or delayed CN can be considered in carefully selected patients with poly-metastatic RCC. Likewise, nephrectomy and complete MDT can be considered before or after IO-based systemic therapy in appropriately selected patients with oligometastatic RCC, potentially improving prognosis.

Statement 6. In oligometastatic RCC, metastasis-directed SBRT may be considered to delay both progression and the need for systemic therapy.

Statement 7. In selected patients with oligometastatic RCC, the following treatment approaches can be considered:

- SBRT followed by active surveillance;
- SBRT followed by IO+TKI; and
- SBRT followed by IO+IO.

The potential of SBRT in treating mRCC (with or without prior systemic therapy) was shown in phase 2 single-arm studies (94, 95). In the oligometastatic setting, SBRT is a feasible treatment option in selected patients who are likely to delay the initiation of systemic therapy or even be cured using local therapy (10, 96). In a phase 2 single-arm trial (97), patients who received SBRT to oligometastases (≤ 5 lesions) from clear cell RCC post nephrectomy had a median PFS of 22.7 months (1-year PFS rate, 64%) at a median follow-up of 17.5 months, and an 82% rate of systemic therapy-free survival at 1 year. A phase 2 single-arm trial of systemic therapy-naïve patients with oligometastatic RCC (≤ 3 extracranial lesions) showed that metastasis-directed SBRT yielded a 91.3% rate of freedom from systemic therapy, an 82.6% rate of PFS, and a 100% rate of local control at 1 year (98). Major limitations of these studies included small sample sizes and the absence of comparators.

Retrospective data from routine clinical practice (99, 100) also demonstrate that SBRT offers an excellent local control rate (up to 98% at 3 years) and a favourable safety profile in patients with oligometastatic RCC (1–5 lesions). Additionally, patients with metachronous disease, solitary metastasis, or non-bone metastasis may have a prolonged interval free from systemic therapy following SBRT (99), whereas patients with bone or brain metastases, or a higher number of lesions (≥ 2), may benefit from upfront systemic therapy rather than sequential SBRT (100).

In a meta-analysis of 28 studies (mostly retrospective) on SBRT for oligometastatic RCC (regardless of *de novo*, metachronous, or oligoprogressive setting), patients with extracranial disease achieved a 90% rate of local control, an 87% rate of OS, and a 0.7% incidence of grade 3/4 toxicities at 1 year, while the corresponding figures were 90%, 50%, and 1.1%, respectively, in patients with intracranial disease (101).

In the phase 1/2 single-arm RAPPORT trial (102), SBRT to all metastatic sites (including the adrenal glands, bone, lungs, lymph nodes, and soft tissue) followed by eight-cycle pembrolizumab therapy demonstrated promising safety outcomes (grade 3 toxicities, 13%; no grade 4/5 toxicities), good local disease control (92% free from local progression at 2 years), and favourable PFS (60% at 1 year; 45% at 2 years) among patients (N = 30) who had received ≤ 2 lines of systemic therapy (77% treatment-naïve) and had 1–5 (median 3) metastatic lesions from clear cell RCC.

Ongoing studies are investigating SBRT to the primary kidney tumour combined with IO-based systemic therapy in patients with newly diagnosed poly-metastatic RCC (103, 104). Concomitant use of SBRT and systemic therapy in synchronous and metachronous oligometastatic RCC will be evaluated in the phase 3 randomised EA8211-SOAR trial (systemic therapy alone as comparator) (105) and the phase 2 randomised PE-PE study (pembrolizumab followed by MDT [SBRT or metastasectomy] vs. MDT alone) (106).

The panel agreed that SBRT followed by active surveillance or IO-based systemic therapy is viable in patients with oligometastatic RCC. Considering the absence of SBRT in KEYNOTE-564 (6, 27), no consensus on adopting SBRT plus adjuvant pembrolizumab was reached.

#### 3.5.3 Management of oligorecurrent/oligoprogressive RCC

Statement 8. In patients with oligorecurrent disease after nephrectomy:

- Systemic IO-based combination therapies should be the first-line treatment.
- MDT (e.g. SBRT) can be considered to delay progression.

In general, patients with oligorecurrent RCC following nephrectomy may have more aggressive and rapidly progressive disease that prompts the need for upfront systemic therapy. Concomitant MDT can also be considered, potentially offering synergistic effects to improve disease control.

The panel did not reach consensus on the management approach for oligorecurrent disease in the post-adjuvant pembrolizumab setting. However, referring to Part 3.2.2, upfront systemic therapy is deemed necessary for poly-metastatic recurrence following adjuvant therapy, and the prior response to adjuvant therapy may be considered for treatment selection.

Statement 9. In patients with oligoprogressive metastases following nephrectomy and systemic IO-based therapy:

- SBRT to the new lesions followed by continuation of the current systemic therapy should be considered.
- MDT typically requires withholding IO/TKI therapy temporarily.

In the oligoprogressive setting, SBRT, in combination with the existing systemic therapy, can be considered to delay the initiation of next-line systemic treatment (96). An expert panel from the European Society for Radiotherapy and Oncology (107) achieved consensus (≥ 80% agreement) on using SBRT in patients with ≤ 3 lesions in the oligoprogressive setting, regardless of patient age, histology, or metastatic site. Other consensus points included: SBRT for bone oligometastases regardless of site; SBRT as the first-line treatment option for adrenal oligometastases; and administration of IO concomitantly with SBRT (within 30 days of SBRT) (107).

A phase 2 single-arm trial (108) evaluated 20 patients with mRCC (excluding IMDC high risk) on first- to fourth-line systemic therapy, including IO-based combinations. Eligible patients had ≤ 3 sites of progression (including new sites) involving ≤ 30% of all sites. SBRT to oligoprogressive lesions at the outset longitudinally prolonged the duration of ongoing systemic therapy by > 6 months in 70% of patients (95% CI, 49.9–90.1) at a median follow-up of 10.4 months. The median time from SBRT to the onset of new systemic therapy or death was 11.1 (95% CI, 4.5–19.3) months. In another phase 2 single-arm study (109), concomitant use of SBRT maintained the original TKI therapy for a median of 12.6 months in 37 patients who developed oligoprogression (≤ 5 metastases) during TKI therapy. Multiple retrospective studies (110–114) similarly revealed that SBRT, with continuation of the current TKI therapy, was well-tolerated and effective at delaying the initiation of subsequent systemic treatment. Furthermore, it may prolong OS compared with continuing TKI alone in patients with oligoprogression. However, major pitfalls of these studies include small sample sizes and a lack of comparators. Prospective trials are underway to investigate the concurrent use of SBRT with IO-based combination therapy or TKI monotherapy in patients who have developed oligoprogression following such systemic treatments (115).

The panel agreed that patients with oligoprogressive RCC following nephrectomy and systemic therapy can be managed by temporarily withholding systemic therapy, initiating SBRT to the new/progressive lesions, and eventually resuming the original regimen. However, they did not reach consensus on whether to use metastasectomy instead of SBRT or to switch directly to next-line systemic therapy.

Despite reviewing multiple studies on the effectiveness and safety of thermal ablation techniques (e.g. cryoablation, radiofrequency ablation, and microwave ablation) in patients with oligometastatic RCC (11–13), the panel did not reach consensus on its role in treating oligometastatic or oligoprogressive RCC. These studies were limited by retrospective design, small patient numbers, short follow-up periods, and the absence of comparators (e.g. metastasectomy or SBRT).

## 4 Discussion and conclusion

The HKUA and the HKSUO convened an expert panel to update consensus statements on managing la/mRCC, drawing on recent clinical evidence and insights from an extensive literature review and panel discussions. Compared with the previous version, the updated consensus statements introduce important changes in several areas: adjuvant therapy for laRCC, active surveillance for mRCC patients with a very favourable prognosis; broader use of IO-based combination therapy across mRCC subtypes; incorporation of the novel therapeutic agent belzutifan; and refined definitions and management strategies for oligometastatic or oligoprogressive RCC. However, a key limitation is that many recommendations are based on retrospective data, indirect comparisons, and expert consensus, rather than on high-level prospective evidence. These consensus statements require ongoing review and updates to address several remaining research gaps: the role of CN (particularly consolidative CN following IO-based systemic therapy), optimal therapy for metastatic recurrence following adjuvant pembrolizumab, belzutifan-based combination therapies, targeted therapies for non-clear cell RCC subtypes, and optimal use of SBRT and metastasectomy (with or without IO-based systemic therapy) in oligometastatic or oligoprogressive RCC. While certain areas warrant further investigation, these consensus statements encapsulate contemporary practices in the management of la/mRCC, aiming to serve as practical references for clinicians in Hong Kong and across the region.
