# Supplementary material for: Hong Kong Urological Association–Hong Kong Society of Uro-Oncology 2025 consensus on the management of locally advanced or metastatic renal cell carcinoma
Source: Front Oncol. 2026 Apr 23;16:1799885. doi: 10.3389/fonc.2026.1799885 (PMC13149114; doi:10.3389/fonc.2026.1799885)
Supplement: Supplementary file 1 [file Table1.docx]

Appendix S1. Full voting records for all accepted and rejected statements.

| **Part 1. Surgical and (neo)adjuvant treatment in locally advanced renal cell carcinoma (RCC)** | | | | | | | | | |
| --- | --- | --- | --- | --- | --- | --- | --- | --- | --- |
| **1.1. Role of cytoreductive nephrectomy (CN)** | | **Response options* (%)** | | | | | **Statement was accepted only if  (A + B)% ≥ 80%** | | |
| **#** | **Drafted statements** | **A** | **B** | **C** | **D** | **E** | **A + B (%)** | **Accepted** | **Voted down** |
| **1** | Upfront CN can be considered in patients with: |  | | | | | | | |
| **1a** | Advanced age (≥ 65 years) | 43% | 43% | 0% | 7% | 7% | 86% | ✔ |  |
| **1b** | Poor performance status (Eastern Cooperative Oncology Group [ECOG] ≥ 2) | 14% | 14% | 29% | 14% | 29% | 29% |  | ✔ |
| **1c** | Severe kidney-related symptoms (e.g. severe haematuria) | 57% | 36% | 0% | 0% | 7% | 93% | ✔ |  |
| **1d** | International Metastatic RCC Database Consortium (IMDC) intermediate-risk disease | 29% | 36% | 21% | 14% | 0% | 64% |  | ✔ |
| **1e** | IMDC poor-risk disease | 7% | 7% | 14% | 21% | 50% | 14% |  | ✔ |
| **1f** | Small primary tumour (i.e. ≤ 4 cm) | 50% | 29% | 14% | 0% | 7% | 79% |  | ✔ |
| **1g** | Oligometastases or low metastatic burden | 64% | 21% | 7% | 7% | 0% | 86% | ✔ |  |
| **1h** | Sarcomatoid features | 14% | 43% | 21% | 14% | 7% | 57% |  | ✔ |
| **1i** | Non-clear cell (ncc)RCC | 29% | 29% | 36% | 7% | 0% | 57% |  | ✔ |
| **1j** | Rapidly progressive disease | 0% | 7% | 21% | 29% | 43% | 7% |  | ✔ |
| **2** | Deferred CN can be considered in selected patients who have received: |  | | | | | | | |
| **2a** | Immunotherapy (IO)-based combination therapy (IO+IO or IO + tyrosine kinase inhibitor [TKI]) | 57% | 36% | 7% | 0% | 0% | 93% | ✔ |  |
| **2b** | TKI-based therapy | 43% | 50% | 7% | 0% | 0% | 93% | ✔ |  |
|  |  |  |  |  |  |  | **Total:** | **5** | **7** |
| *Response options include A: accept completely; B: accept with some reservation; C: accept with major reservation; D: reject with reservation; and E: reject completely. | | | | | | | | | |

| **Part 1. Surgical and (neo)adjuvant treatment in locally advanced RCC** | | | | | | | | | |
| --- | --- | --- | --- | --- | --- | --- | --- | --- | --- |
| **1.2. Role of neoadjuvant therapy** | | **Response options* (%)** | | | | | **Statement was accepted only if  (A + B)% ≥ 80%** | | |
| **#** | **Drafted statements** | **A** | **B** | **C** | **D** | **E** | **A + B (%)** | **Accepted** | **Voted down** |
| **3** | Routine use of neoadjuvant systemic therapy for locally advanced RCC is not recommended. | 57% | 43% | 0% | 0% | 0% | 100% | ✔ |  |
|  |  |  |  |  |  |  | **Total:** | **1** | **0** |
| *Response options include A: accept completely; B: accept with some reservation; C: accept with major reservation; D: reject with reservation; and E: reject completely. | | | | | | | | | |

| **Part 1. Surgical and (neo)adjuvant treatment in locally advanced RCC** | | | | | | | | | |
| --- | --- | --- | --- | --- | --- | --- | --- | --- | --- |
| **1.3. Role of adjuvant therapy** | | **Response options* (%)** | | | | | **Statement was accepted only if  (A + B)% ≥ 80%** | | |
| **#** | **Drafted statements** | **A** | **B** | **C** | **D** | **E** | **A + B (%)** | **Accepted** | **Voted down** |
| **4** | Urologists should serve as a care coordinator and refer those with intermediate-to-high risk of recurrence and M1 with no evidence of disease (NED) to an oncologist to assess the need for adjuvant therapy. | 93% | 0% | 7% | 0% | 0% | 93% | ✔ |  |
| **5** | Adjuvant therapy should be initiated within 16 weeks after nephrectomy for eligible patients. | 71% | 29% | 0% | 0% | 0% | 100% | ✔ |  |
|  |  |  |  |  |  |  | **Total:** | **2** | **0** |
| *Response options include A: accept completely; B: accept with some reservation; C: accept with major reservation; D: reject with reservation; and E: reject completely. | | | | | | | | | |

| **Part 1. Surgical and (neo)adjuvant treatment in locally advanced RCC** | | | | | | | | | |
| --- | --- | --- | --- | --- | --- | --- | --- | --- | --- |
| **1.4. Monitoring of adjuvant therapy** | | **Response options* (%)** | | | | | **Statement was accepted only if  (A + B)% ≥ 80%** | | |
| **#** | **Drafted statements** | **A** | **B** | **C** | **D** | **E** | **A + B (%)** | **Accepted** | **Voted down** |
| **6** | Before the start of adjuvant therapy, contrast-enhanced computed tomography (CT) of the chest, abdomen, and pelvis is recommended. | 86% | 14% | 0% | 0% | 0% | 100% | ✔ |  |
| **7** | During adjuvant therapy, complete blood counts, liver function, and creatinine and thyroid-stimulating hormone levels should be checked regularly. | 100% | 0% | 0% | 0% | 0% | 100% | ✔ |  |
| **8** | In years 1 and 2 post adjuvant therapy, contrast-enhanced CT of the chest, abdomen, and pelvis every 3–6 months is recommended. | 64% | 36% | 0% | 0% | 0% | 100% | ✔ |  |
| **9** | In years 3–5 post adjuvant therapy, contrast-enhanced CT of the chest, abdomen, and pelvis every 6 months is recommended. | 57% | 36% | 7% | 0% | 0% | 93% | ✔ |  |
| **10** | In cases of early emergence of new lesions on imaging, a biopsy should be considered to rule out pseudo-progression. | 29% | 50% | 14% | 7% | 0% | 79% |  | ✔ |
| **11** | After year 5 post adjuvant therapy, the frequency of imaging and duration of follow-up should be tailored to each patient’s risk of recurrence. | 93% | 7% | 0% | 0% | 0% | 100% | ✔ |  |
| **12** | Positron emission tomography (PET)-CT or magnetic resonance imaging (MRI) can be considered to assess cases with equivocal CT findings. | 79% | 21% | 0% | 0% | 0% | 100% | ✔ |  |
| **13** | MRI can be used if CT is contraindicated. | 86% | 14% | 0% | 0% | 0% | 100% | ✔ |  |
| **14** | The following imaging is recommended only when clinically indicated: |  | | | | | | | |
| **14a** | Imaging of the bone | 57% | 43% | 0% | 0% | 0% | 100% | ✔ |  |
| **14b** | Imaging of the brain | 50% | 50% | 0% | 0% | 0% | 100% | ✔ |  |
|  |  |  |  |  |  |  | **Total:** | **9** | **1** |
| *Response options include A: accept completely; B: accept with some reservation; C: accept with major reservation; D: reject with reservation; and E: reject completely. | | | | | | | | | |

| **Part 2. First-line management of metastatic clear cell RCC (mccRCC)** | | | | | | | | | |
| --- | --- | --- | --- | --- | --- | --- | --- | --- | --- |
| **2.1. Role of genomic testing** | | **Response options* (%)** | | | | | **Statement was accepted only if  (A + B)% ≥ 80%** | | |
| **#** | **Drafted statements** | **A** | **B** | **C** | **D** | **E** | **A + B (%)** | **Accepted** | **Voted down** |
| **1** | Routine genomic testing to inform treatment selection for mccRCC is not recommended. | 64% | 29% | 7% | 0% | 0% | 93% | ✔ |  |
|  |  |  |  |  |  |  | **Total:** | **1** | **0** |
| *Response options include A: accept completely; B: accept with some reservation; C: accept with major reservation; D: reject with reservation; and E: reject completely. | | | | | | | | | |

| **Part 2. First-line management of mccRCC** | | | | | | | | | |
| --- | --- | --- | --- | --- | --- | --- | --- | --- | --- |
| **2.2. Management of patients with prior exposure to adjuvant pembrolizumab** | | **Response options* (%)** | | | | | **Statement was accepted only if  (A + B)% ≥ 80%** | | |
| **#** | **Drafted statements** | **A** | **B** | **C** | **D** | **E** | **A + B (%)** | **Accepted** | **Voted down** |
| **2** | In patients who have received adjuvant pembrolizumab, IO+IO is not recommended in the metastatic setting. | 14% | 43% | 7% | 36% | 0% | 57% |  | ✔ |
| **3** | In patients who have metastatic recurrence during adjuvant pembrolizumab: |  | | | | | | | |
| **3a** | TKI monotherapy is a treatment option. | 57% | 43% | 0% | 0% | 0% | 100% | ✔ |  |
| **3b** | The immediate initiation of IO+TKI is not recommended. | 29% | 36% | 14% | 21% | 0% | 64% |  | ✔ |
| **4** | Rechallenging with IO+TKI may be considered in the metastatic setting in: |  | | | | | | | |
| **4a** | Patients who have recurrence ≥ 6 months after the completion of adjuvant pembrolizumab. | 21% | 50% | 21% | 7% | 0% | 71% |  | ✔ |
| **4b** | Patients who have recurrence ≥ 12 months after the completion of adjuvant pembrolizumab. | 57% | 36% | 7% | 0% | 0% | 93% | ✔ |  |
|  |  |  |  |  |  |  | **Total:** | **2** | **3** |
| *Response options include A: accept completely; B: accept with some reservation; C: accept with major reservation; D: reject with reservation; and E: reject completely. | | | | | | | | | |

| **Part 2. First-line management of mccRCC** | | | | | | | | | |
| --- | --- | --- | --- | --- | --- | --- | --- | --- | --- |
| **2.3. Selection of first-line systemic therapy** | | **Response options* (%)** | | | | | **Statement was accepted only if  (A + B)% ≥ 80%** | | |
| **#** | **Drafted statements** | **A** | **B** | **C** | **D** | **E** | **A + B (%)** | **Accepted** | **Voted down** |
| **5** | Active surveillance is a feasible management approach for selected patients (e.g. IMDC very-favourable risk, oligometastatic disease [refer to Part 5], low metastatic volume, or compliance with a follow-up schedule). | 57% | 29% | 7% | 7% | 0% | 86% | ✔ |  |
| **6** | In patients with IMDC very-favourable risk, TKI monotherapy is a treatment option. | 57% | 43% | 0% | 0% | 0% | 100% | ✔ |  |
| **7** | IO+TKI is the standard of care for IMDC favourable-risk patients. | 64% | 29% | 0% | 7% | 0% | 93% | ✔ |  |
| **8** | TKI monotherapy is an alternative regimen for selected patients with IMDC favourable risk (e.g. low metastatic burden and no symptoms). | 64% | 36% | 0% | 0% | 0% | 100% | ✔ |  |
| **9** | IMDC intermediate/poor-risk patients should be treated with IO+IO (30) or IO+TKI. | 100% | 0% | 0% | 0% | 0% | 100% | ✔ |  |
| **10** | IO+TKI is the preferred regimen for IMDC intermediate/poor-risk patients with rapidly progressive or extensive disease. | 71% | 21% | 7% | 0% | 0% | 93% | ✔ |  |
| **11** | Treatment discontinuation can be considered in: |  | | | | | | | |
| **11a** | Patients who have completed 2 years of IO+TKI treatment. | 21% | 57% | 21% | 0% | 0% | 79% |  | ✔ |
| **11b** | Patients who have completed 2 years of IO+IO treatment. | 36% | 50% | 14% | 0% | 0% | 86% | ✔ |  |
|  |  |  |  |  |  |  | **Total:** | **7** | **1** |
| *Response options include A: accept completely; B: accept with some reservation; C: accept with major reservation; D: reject with reservation; and E: reject completely. | | | | | | | | | |

| **Part 2. First-line management of mccRCC** | | | | | | | | | |
| --- | --- | --- | --- | --- | --- | --- | --- | --- | --- |
| **2.4. Treatment monitoring** | | **Response options* (%)** | | | | | **Statement was accepted only if  (A + B)% ≥ 80%** | | |
| **#** | **Drafted statements** | **A** | **B** | **C** | **D** | **E** | **A + B (%)** | **Accepted** | **Voted down** |
| **12** | To monitor treatment response, contrast-enhanced CT of the thorax, abdomen, and pelvis should be performed regularly (e.g. every 2–4 months. | 71% | 29% | 0% | 0% | 0% | 100% | ✔ |  |
| **13** | Imaging of regions outside the chest, abdomen, and pelvis should only be performed when clinically indicated. | 100% | 0% | 0% | 0% | 0% | 100% | ✔ |  |
| **14** | In patients who have stopped treatment (e.g. those with a complete response), imaging can be performed when clinically indicated. | 50% | 29% | 14% | 7% | 0% | 79% |  | ✔ |
|  |  |  |  |  |  |  | **Total:** | **2** | **1** |
| *Response options include A: accept completely; B: accept with some reservation; C: accept with major reservation; D: reject with reservation; and E: reject completely. | | | | | | | | | |

| **Part 3. Second- and later-line treatment of mccRCC** | | | | | | | | | |
| --- | --- | --- | --- | --- | --- | --- | --- | --- | --- |
| **3.1. Second-line treatment** | | **Response options* (%)** | | | | | **Statement was accepted only if  (A + B)% ≥ 80%** | | |
| **#** | **Drafted statements** | **A** | **B** | **C** | **D** | **E** | **A + B (%)** | **Accepted** | **Voted down** |
| **1** | IO rechallenge is not recommended in patients who have progressed on IO+IO or IO+TKI in the first-line metastatic setting. | 57% | 43% | 0% | 0% | 0% | 100% | ✔ |  |
| **2** | In patients who have progressed on IO+IO or IO+TKI in the first-line metastatic setting, subsequent treatment options include: |  | | | | | | | |
| **2a** | Any TKI that has not previously been used | 71% | 29% | 0% | 0% | 0% | 100% | ✔ |  |
| **2b** | Lenvatinib + everolimus | 50% | 43% | 0% | 7% | 0% | 93% | ✔ |  |
| **2c** | Lenvatinib + belzutifan | 57% | 36% | 0% | 7% | 0% | 93% | ✔ |  |
| **3** | Belzutifan is a treatment option in patients who have been exposed to a TKI and an IO (in combination or sequentially). | 86% | 14% | 0% | 0% | 0% | 100% | ✔ |  |
| **4** | Patients who have progressed on first-line TKI monotherapy can be treated with: |  | | | | | | | |
| **4a** | Cabozantinib. | 64% | 36% | 0% | 0% | 0% | 100% | ✔ |  |
| **4b** | Nivolumab. | 86% | 14% | 0% | 0% | 0% | 100% | ✔ |  |
| **4c** | Lenvatinib + everolimus. | 50% | 36% | 7% | 7% | 0% | 86% | ✔ |  |
| **4d** | IO+TKI. | 50% | 43% | 7% | 0% | 0% | 93% | ✔ |  |
|  |  |  |  |  |  |  | **Total:** | **9** | **0** |
| *Response options include A: accept completely; B: accept with some reservation; C: accept with major reservation; D: reject with reservation; and E: reject completely. | | | | | | | | | |

| **Part 3. Second- and later-line treatment of mccRCC** | | | | | | | | | |
| --- | --- | --- | --- | --- | --- | --- | --- | --- | --- |
| **3.2. Third-line treatment and beyond** | | **Response options* (%)** | | | | | **Statement was accepted only if  (A + B)% ≥ 80%** | | |
| **#** | **Drafted statements** | **A** | **B** | **C** | **D** | **E** | **A + B (%)** | **Accepted** | **Voted down** |
| **5** | In patients exposed to an IO, a TKI, and belzutifan, lenvatinib + everolimus may be a treatment option. | 57% | 36% | 7% | 0% | 0% | 93% | ✔ |  |
|  |  |  |  |  |  |  | **Total:** | **1** | **0** |
| *Response options include A: accept completely; B: accept with some reservation; C: accept with major reservation; D: reject with reservation; and E: reject completely. | | | | | | | | | |

| **Part 4. Management of metastatic (m)RCC with sarcomatoid features or ncc histology** | | | | | | | | | |
| --- | --- | --- | --- | --- | --- | --- | --- | --- | --- |
| **4.1. Management of patients with sarcomatoid features** | | **Response options* (%)** | | | | | **Statement was accepted only if  (A + B)% ≥ 80%** | | |
| **#** | **Drafted statements** | **A** | **B** | **C** | **D** | **E** | **A + B (%)** | **Accepted** | **Voted down** |
| **1** | IO+IO or IO+TKI is recommended for IMDC intermediate/poor-risk patients with mRCC and sarcomatoid dedifferentiation. | 93% | 7% | 0% | 0% | 0% | 100% | ✔ |  |
| **2** | In IMDC favourable-risk patients with mRCC and sarcomatoid dedifferentiation: |  | | | | | | | |
| **2a** | IO+IO is a treatment option. | 43% | 57% | 0% | 0% | 0% | 100% | ✔ |  |
| **2b** | IO+TKI is a treatment option. | 64% | 36% | 0% | 0% | 0% | 100% | ✔ |  |
|  |  |  |  |  |  |  | **Total:** | **3** | **0** |
| *Response options include A: accept completely; B: accept with some reservation; C: accept with major reservation; D: reject with reservation; and E: reject completely. | | | | | | | | | |

| **Part 4. Management of mRCC with sarcomatoid features or ncc histology** | | | | | | | | | |
| --- | --- | --- | --- | --- | --- | --- | --- | --- | --- |
| **4.2. Management of patients with nccRCC** | | **Response options* (%)** | | | | | **Statement was accepted only if  (A + B)% ≥ 80%** | | |
| **#** | **Drafted statements** | **A** | **B** | **C** | **D** | **E** | **A + B (%)** | **Accepted** | **Voted down** |
| **3** | Preferred first-line treatment options for advanced nccRCC include: |  | | | | | | | |
| **3a** | Pembrolizumab + lenvatinib. | 86% | 14% | 0% | 0% | 0% | 100% | ✔ |  |
| **3b** | Pembrolizumab + axitinib. | 29% | 21% | 36% | 14% | 0% | 50% |  | ✔ |
| **3c** | Nivolumab + cabozantinib. | 50% | 50% | 0% | 0% | 0% | 100% | ✔ |  |
| **3d** | Nivolumab + ipilimumab. | 43% | 50% | 0% | 7% | 0% | 93% | ✔ |  |
| **3e** | Cabozantinib. | 50% | 36% | 7% | 7% | 0% | 86% | ✔ |  |
|  |  |  |  |  |  |  | **Total:** | **4** | **1** |
| *Response options include A: accept completely; B: accept with some reservation; C: accept with major reservation; D: reject with reservation; and E: reject completely. | | | | | | | | | |

| **Part 5. Management of oligometastatic RCC** | | | | | | | | | |
| --- | --- | --- | --- | --- | --- | --- | --- | --- | --- |
| **5.1. Definitions of oligometastatic and oligoprogressive RCC** | | **Response options* (%)** | | | | | **Statement was accepted only if  (A + B)% ≥ 80%** | | |
| **#** | **Drafted statements** | **A** | **B** | **C** | **D** | **E** | **A + B (%)** | **Accepted** | **Voted down** |
| **1** | Synchronous oligometastatic RCC should be defined as: |  | | | | | | | |
| **1a** | ≤ 3 metastatic lesions on contrast-enhanced CT at initial diagnosis. | 64% | 21% | 0% | 14% | 0% | 86% | ✔ |  |
| **1b** | ≤ 5 metastatic lesions on contrast-enhanced CT at initial diagnosis. | 36% | 36% | 7% | 14% | 7% | 71% |  | ✔ |
| **2** | Metachronous oligometastatic RCC should be defined as: |  | | | | | | | |
| **2a** | ≤ 3 metastatic lesions on contrast-enhanced CT > 1 year after primary tumour diagnosis. | 57% | 29% | 0% | 14% | 0% | 86% | ✔ |  |
| **2b** | ≤ 5 metastatic lesions on contrast-enhanced CT > 1 year after primary tumour diagnosis. | 29% | 43% | 7% | 14% | 7% | 71% |  | ✔ |
| **3** | Oligoprogressive RCC should be defined as: |  | | | | | | | |
| **3a** | ≤ 3 new/progressive metastatic lesions on contrast-enhanced CT following systemic therapy. | 71% | 14% | 0% | 14% | 0% | 86% | ✔ |  |
| **3b** | ≤ 5 new/progressive metastatic lesions on contrast-enhanced CT following systemic therapy. | 29% | 43% | 7% | 14% | 7% | 71% |  | ✔ |
| **3c** | Enlargement by Response Evaluation Criteria in Solid Tumours of known metastatic lesions on contrast-enhanced CT following systemic therapy. | 57% | 36% | 7% | 0% | 0% | 93% | ✔ |  |
|  |  |  |  |  |  |  | **Total:** | **4** | **3** |
| *Response options include A: accept completely; B: accept with some reservation; C: accept with major reservation; D: reject with reservation; and E: reject completely. | | | | | | | | | |

| **Part 5. Management of oligometastatic RCC** | | | | | | | | | |
| --- | --- | --- | --- | --- | --- | --- | --- | --- | --- |
| **5.2. Management of oligometastatic RCC** | | **Response options* (%)** | | | | | **Statement was accepted only if  (A + B)% ≥ 80%** | | |
| **#** | **Drafted statements** | **A** | **B** | **C** | **D** | **E** | **A + B (%)** | **Accepted** | **Voted down** |
| **4** | Upfront systemic therapy, rather than metastasis-directed therapy, is preferable in patients with oligometastatic RCC who have: |  | | | | | | | |
| **4a** | IMDC intermediate risk. | 36% | 36% | 14% | 14% | 0% | 71% |  | ✔ |
| **4b** | IMDC poor risk. | 79% | 21% | 0% | 0% | 0% | 100% | ✔ |  |
| **4c** | Poor ECOG performance status (≥ 2). | 43% | 36% | 14% | 7% | 0% | 79% |  | ✔ |
| **4d** | Brain metastases. | 36% | 57% | 0% | 7% | 0% | 93% | ✔ |  |
| **4e** | Bone metastases. | 29% | 43% | 14% | 14% | 0% | 71% |  | ✔ |
| **4f** | nccRCC. | 21% | 64% | 7% | 7% | 0% | 86% | ✔ |  |
| **4g** | Sarcomatoid features. | 50% | 43% | 7% | 0% | 0% | 93% | ✔ |  |
| **4h** | Rapidly progressive disease within 6 months. | 79% | 21% | 0% | 0% | 0% | 100% | ✔ |  |
| **5** | In selected patients with oligometastatic RCC, viable treatment options include: |  | | | | | | | |
| **5a** | Nephrectomy and complete metastasis-directed therapy (MDT) followed by active surveillance. | 43% | 43% | 14% | 0% | 0% | 86% | ✔ |  |
| **5b** | Nephrectomy and complete MDT followed by adjuvant pembrolizumab. | 57% | 43% | 0% | 0% | 0% | 100% | ✔ |  |
| **5c** | Nephrectomy and complete MDT followed by IO combinations. | 29% | 57% | 14% | 0% | 0% | 86% | ✔ |  |
| **5d** | IO-based combination therapy followed by consolidative nephrectomy and MDT in good responders. | 50% | 50% | 0% | 0% | 0% | 100% | ✔ |  |
| **6** | In oligometastatic RCC, metastasis-directed stereotactic body radiotherapy (SBRT) may be considered to delay both progression and the need for systemic therapy. | 57% | 36% | 7% | 0% | 0% | 93% | ✔ |  |
| **7** | In selected patients with oligometastatic RCC, the following treatment approaches can be considered: |  | | | | | | | |
| **7a** | SBRT followed by active surveillance. | 43% | 43% | 14% | 0% | 0% | 86% | ✔ |  |
| **7b** | SBRT followed by adjuvant pembrolizumab. | 29% | 43% | 21% | 7% | 0% | 71% |  | ✔ |
| **7c** | SBRT followed by IO+TKI. | 7% | 79% | 14% | 0% | 0% | 86% | ✔ |  |
| **7d** | SBRT followed by IO+IO. | 14% | 79% | 7% | 0% | 0% | 93% | ✔ |  |
|  |  |  |  |  |  |  | **Total:** | **13** | **4** |
| *Response options include A: accept completely; B: accept with some reservation; C: accept with major reservation; D: reject with reservation; and E: reject completely. | | | | | | | | | |

| **Part 5. Management of oligometastatic RCC** | | | | | | | | | |
| --- | --- | --- | --- | --- | --- | --- | --- | --- | --- |
| **5.3. Management of oligorecurrent / oligoprogressive RCC** | | **Response options* (%)** | | | | | **Statement was accepted only if  (A + B)% ≥ 80%** | | |
| **#** | **Drafted statements** | **A** | **B** | **C** | **D** | **E** | **A + B (%)** | **Accepted** | **Voted down** |
| **8** | In patients with oligorecurrent disease after nephrectomy: |  | | | | | | | |
| **8a** | Systemic IO-based combination therapies should be the first-line treatment. | 50% | 50% | 0% | 0% | 0% | 100% | ✔ |  |
| **8b** | MDT (e.g. SBRT) can be considered to delay progression. | 50% | 43% | 7% | 0% | 0% | 93% | ✔ |  |
| **8c** | For early-to-intermediate recurrence occurring < 6 months after adjuvant pembrolizumab completion, TKI monotherapy can be considered. | 57% | 21% | 14% | 7% | 0% | 79% |  | ✔ |
| **8d** | For late recurrence occurring ≥ 6 months after adjuvant pembrolizumab completion, IO+TKI combinations can be considered. | 29% | 50% | 21% | 0% | 0% | 79% |  | ✔ |
| **9** | In patients with oligoprogressive metastases following nephrectomy and systemic IO-based therapy: |  | | | | | | | |
| **9a** | Metastasectomy followed by continuation of the current systemic therapy should be considered. | 14% | 57% | 21% | 7% | 0% | 71% |  | ✔ |
| **9b** | SBRT to the new lesions followed by continuation of the current systemic therapy should be considered. | 29% | 57% | 14% | 0% | 0% | 86% | ✔ |  |
| **9c** | Thermal ablation (e.g. radiofrequency ablation, cryoablation, and microwave ablation) should be considered. | 14% | 50% | 21% | 7% | 7% | 64% |  | ✔ |
| **9d** | MDT typically requires withholding IO/TKI therapy temporarily. | 21% | 64% | 7% | 7% | 0% | 86% | ✔ |  |
| **9e** | Changing the lines of systemic therapy should be considered. | 36% | 43% | 14% | 7% | 0% | 79% |  | ✔ |
|  |  |  |  |  |  |  | **Total:** | **4** | **5** |
| *Response options include A: accept completely; B: accept with some reservation; C: accept with major reservation; D: reject with reservation; and E: reject completely. | | | | | | | | | |
