# Supplementary material for: Hong Kong Urological Association–Hong Kong Society of Uro-Oncology 2025 consensus on the management of locally advanced or metastatic renal cell carcinoma
Source: Front Oncol. 2026 Apr 23;16:1799885. doi: 10.3389/fonc.2026.1799885 (PMC13149114; doi:10.3389/fonc.2026.1799885)
Supplement: Supplementary file 2 [file Table2.docx]

Appendix S2 – Prophylactic and supportive approaches for common side effects related to immunotherapy (IO) and tyrosine kinase inhibitors (TKI) (1, 2).

| Culprit agent | Side effect | Prophylaxis | Supportive care |
| --- | --- | --- | --- |
| IO or TKI | Diarrhoea | Consume frequent, small meals  Avoid alcohol and caffeine  Avoid dairy products  Avoid foods that are spicy, fatty, or high in insoluble fibre | Administer antidiarrhoeals (primarily loperamide)  Maintain hydration  Incorporate the BRAT diet: bananas, rice, applesauce, and toast |
| IO or TKI | Fatigue | Maintain adequate diet and fluid intake  Increase activity level, but rest when energy is low  Monitor for thyroid disease and other fatigue-related conditions | Exclude other causes of fatigue  Refer to dietitian  Refer to physiotherapist  Consider psychosocial interventions (e.g. cognitive behavioural therapy)  Consider psychostimulants |
| IO or TKI | Stomatitis | Undergo dental examinations regularly  Avoid alcohol, smoking, and foods that salty, spicy, or acidic  Inspect the mouth daily  Use alcohol-free mouthwash daily  Remove dentures before oral care; dentures should also be brushed with toothpaste  Eliminate ill-fitted prostheses and fractured teeth | Hydrate to keep the mouth moist  Use sodium bicarbonate-containing oral rinses  Treat pain using ice chips, anaesthetic mouthwashes, or systemic analgesics  Treat oral dryness using sugar-free gum or candy, or salivary substitutes |
| IO or TKI | Hand-foot syndrome | Avoid hot water  Avoid friction and pressure on hands and feet  Keep hands and feet moisturised using emollient creams and ammonium lactate  Avoid fragranced or foaming cleaners and alcohol-containing hand sanitisers  Use sunscreen | Soften hyperkeratotic areas using creams  Treat symptoms and lesions using topical analgesics  Consider oral analgesics if topical treatment is ineffective  Refer to dermatologist when necessary |
| Primarily TKI | Hypertension | Assess blood pressure and medical history before treatment initiation  Check for interactions between concomitant medications that may affect blood pressure | Measure blood pressure regularly  Prescribe antihypertensives when clinically indicated  Refer to internist when necessary |
| Primarily IO | Elevated liver enzymes | Assess liver enzymes and medical history before treatment initiation  Check for interactions between concomitant medications that may affect liver enzymes | Undergo liver function tests regularly  Prescribe oral or intravenous steroids when clinically indicated  Refer to hepatologist when necessary |

References

1. Fecher LA, Agarwala SS, Hodi FS, Weber JS. Ipilimumab and its toxicities: a multidisciplinary approach. *Oncologist*. (2013) 18:733-43. doi: 10.1634/theoncologist.2012-0483

2. McGregor B, Mortazavi A, Cordes L, Salabao C, Vandlik S, Apolo AB. Management of adverse events associated with cabozantinib plus nivolumab in renal cell carcinoma: A review. *Cancer Treat Rev*. (2022) 103:102333. doi: 10.1016/j.ctrv.2021.102333
